# Supplementary material for: FreeSurfer version-shuffling can enhance brain age predictions
Source: Neuroimage Rep. 2024 Jul 16;4(3):100214. doi: 10.1016/j.ynirp.2024.100214 (PMC12172796; doi:10.1016/j.ynirp.2024.100214)
Supplement: Multimedia component 1 [file mmc1.docx]

**Supplement**

**Supplement 1**: Training Model Performance Across Algorithms (*initial iteration*)

| **R** | **adjusted R^2^** | **adjusted R^2^ (corrected)** | **MAE** | **RMSE** | **FreeSurfer version** | **Model** |
| --- | --- | --- | --- | --- | --- | --- |
| 0.56 | 0.31 | 0.32 | 4.90 | 6.11 | 5 | XGBoost |
| 0.38 | 0.15 | 0.21 | 5.60 | 6.76 | 5 | SVM |
| 0.60 | 0.36 | 0.36 | 4.80 | 5.90 | 5 | LightGBM |
| 0.65 | 0.42 | 0.42 | 4.52 | 5.58 | 5 | Lasso |
| **0.69** | **0.47** | **0.47** | **4.27** | **5.33** | **5** | **LM** |
| 0.53 | 0.28 | 0.28 | 5.07 | 6.27 | 7 | XGBoost |
| 0.33 | 0.11 | 0.17 | 5.76 | 6.92 | 7 | SVM |
| 0.57 | 0.32 | 0.33 | 4.98 | 6.06 | 7 | LightGBM |
| 0.62 | 0.39 | 0.39 | 4.63 | 5.73 | 7 | Lasso |
| **0.66** | **0.43** | **0.43** | **4.41** | **5.52** | **7** | **LM** |

Modelling Performance Across FreeSurfer Versions and Algorithms in Training Data. R = Pearson’s Correlation coefficient, adjusted R^2^ = adjusted variance explained (linear model), adjusted R^2^ corrected = adjusted variance explained (linear model correcting for sex and scanner site), MAE = mean absolute error, RMSE = root mean squared error. RMSE and MAE are indicated in years.

**Supplement 2**: Test Model Performance Across Algorithms (*initial iteration*)

| **R** | **adjusted R^2^** | **adjusted R^2^ corrected** | **MAE** | **RMSE** | **FreeSurfer version** | **Model** |
| --- | --- | --- | --- | --- | --- | --- |
| 0.58 | 0.34 | 0.34 | 4.78 | 5.95 | FS5toFS5 | XGBoost |
| 0.43 | 0.18 | 0.26 | 5.52 | 6.59 | FS5toFS5 | SVM |
| 0.62 | 0.39 | 0.39 | 4.69 | 5.72 | FS5toFS5 | LightGBM |
| 0.66 | 0.43 | 0.44 | 4.45 | 5.48 | FS5toFS5 | Lasso |
| **0.70** | **0.49** | **0.49** | **4.17** | **5.19** | **FS5toFS5** | **LM** |
| 0.51 | 0.26 | 0.27 | 5.08 | 6.25 | FS5toFS7 | XGBoost |
| 0.36 | 0.13 | 0.21 | 5.72 | 6.86 | FS5toFS7 | SVM |
| 0.56 | 0.31 | 0.32 | 5.03 | 6.09 | FS5toFS7 | LightGBM |
| 0.59 | 0.34 | 0.35 | 4.80 | 5.89 | FS5toFS7 | Lasso |
| **0.63** | **0.40** | **0.41** | **4.69** | **5.80** | **FS5toFS7** | **LM** |
| 0.56 | 0.31 | 0.32 | 5.07 | 6.26 | FS7toFS5 | XGBoost |
| 0.42 | 0.17 | 0.25 | 5.60 | 6.65 | FS7toFS5 | SVM |
| 0.59 | 0.35 | 0.36 | 4.92 | 6.00 | FS7toFS5 | LightGBM |
| 0.64 | 0.41 | 0.41 | 4.52 | 5.63 | FS7toFS5 | Lasso |
| **0.69** | **0.47** | **0.47** | **4.30** | **5.41** | **FS7toFS5** | **LM** |
| 0.55 | 0.31 | 0.31 | 4.92 | 6.08 | FS7toFS7 | XGBoost |
| 0.37 | 0.13 | 0.21 | 5.70 | 6.77 | FS7toFS7 | SVM |
| 0.57 | 0.33 | 0.34 | 4.91 | 5.97 | FS7toFS7 | LightGBM |
| 0.62 | 0.39 | 0.39 | 4.58 | 5.68 | FS7toFS7 | Lasso |
| **0.68** | **0.47** | **0.47** | **4.25** | **5.32** | **FS7toFS7** | **LM** |

Modelling Performance Across FreeSurfer Versions and Algorithms in Test Data. R = Pearson’s Correlation coefficient, adjusted R^2^ = adjusted variance explained (linear model), adjusted R^2^ corrected = adjusted variance explained (linear model correcting for sex and scanner site), MAE = mean absolute error, RMSE = root mean squared error. RMSE and MAE are indicated in years.

**Supplement 3**: Permutation feature importance: top 10 features and their variance explained when predicting age (*initial iteration*)

| **FreeSurfer v5** | **FreeSurfer v7** |
| --- | --- |
| lh_superiorfrontal_area 0.341867 +/- 0.007639  lh_inferiorparietal_area 0.275286 +/- 0.011307  lh_inferiorparietal_volume 0.260479 +/- 0.009025  rh_inferiortemporal_area 0.253444 +/- 0.010406  rh_inferiortemporal_volume 0.243288 +/- 0.007102  lh_superiorfrontal_thickness 0.209069 +/- 0.010630  lh_supramarginal_volume 0.207518 +/- 0.005090  lh_supramarginal_area 0.183548 +/- 0.008399  lh_precentral_volume 0.181147 +/- 0.006269  lh_precentral_area 0.151394 +/- 0.006774 | lh_precentral_volume 0.233111 +/- 0.008399  lh_precentral_area 0.210369 +/- 0.007713  rh_posteriorcingulate_area 0.194168 +/- 0.006988  lh_bankssts_volume 0.165247 +/- 0.005948  lh_bankssts_area 0.162048 +/- 0.010068  lh_medialorbitofrontal_area 0.157978 +/- 0.012232  lh_medialorbitofrontal_volume 0.153799 +/- 0.007422  rh_inferiortemporal_area 0.148451 +/- 0.006095  lh_superiorfrontal_area 0.133032 +/- 0.005665  rh_cuneus_volume 0.120757 +/- 0.005527 |

**Supplement 4: Age stratefied correlations between FS5 and FS7 feature estimates**

Data were grouped into three even bins based on age. This resulted in 3 bins of N = 730 training and N = 735 test data. Mean ages for these bins were M_train 1_ = 56.6, M_train 2_ = 64.6, M_train 3_ = 73.2; and M_test 1_ = 56.8, M_test 2_ = 64.6, M_test 3_ = 73.3.

**Supplement 5: Lasso training and test when 1000 times repeatedly randomly sampling training and test data (compare with Table 1)**

| **Average Training Performance** | | | | |
| --- | --- | --- | --- | --- |
| R | R^2^ | MAE | RMSE | FS version |
| 0.67±.01 | 0.43±.01 | 4.41±.06 | 5.47±.07 | 5 |
| 0.67±.01 | 0.45±.01 | 4.34±.05 | 5.37±.06 | 7 |
| Average Test Performance | | | | |
| R | R^2^ | MAE | RMSE | Modelling |
| 0.63±.01 | 0.38±.02 | 4.62±.07 | 5.73±.08 | FS5 to FS5 |
| 0.62±.01 | 0.38±.01 | 4.62±.05 | 5.72±.06 | FS5 to FS7 |
| 0.62±.01 | 0.38±.01 | 4.62±.05 | 5.72±.06 | FS7 to FS7 |
| 0.62±.01 | 0.38±.02 | 4.62±.07 | 5.73±.08 | FS7 to FS5 |

**Supplement 6: Performance of models varying both training and test splits as well as feature composition (FreeSurfer version)**

|  | **R2** | **r** | **MAE** | **RMSE** | **AIC** | **BIC** |
| --- | --- | --- | --- | --- | --- | --- |
| Mean train | 0.548 | 0.740 | 3.919 | 4.890 | 7384.931 | 8546.773 |
| SD train | 0.010 | 0.007 | 0.050 | 0.058 | 52.120 | 52.120 |
| Mean test | 0.449 | 0.674 | 4.324 | 5.395 | 7813.901 | 8975.650 |
| SD test | 0.013 | 0.008 | 0.054 | 0.063 | 51.580 | 51.580 |

**We used i = 1,000 iterations to randomly split the data into equal parts of training and test data and each of these portions into equal parts of FS5 and FS7 features for training and testing.**
